# Magnetic nanoparticles for oligodendrocyte precursor cell transplantation therapies: progress and challenges

**DOI:** 10.1186/2052-8426-2-23

**Published:** 2014-07-28

**Authors:** Stuart I Jenkins, Humphrey H P Yiu, Matthew J Rosseinsky, Divya M Chari

**Affiliations:** Cellular and Neural Engineering Group, Institute for Science and Technology in Medicine Keele University, Stoke-on-Trent, Staffordshire ST5 5BG UK; School of Engineering and Physical Sciences, Heriot-Watt University, Edinburgh, EH14 4AS UK; Department of Chemistry, University of Liverpool, Liverpool, L69 7ZD UK

**Keywords:** OPC, Uptake, Labeling, Tracking, Iron oxide, Magnetic targeting, Neural cell, Cell therapy

## Abstract

Oligodendrocyte precursor cells (OPCs) have shown high promise as a transplant population to promote regeneration in the central nervous system, specifically, for the production of myelin – the protective sheath around nerve fibers. While clinical trials for these cells have commenced in some areas, there are currently key barriers to the translation of neural cell therapies. These include the ability to (a) image transplant populations *in vivo*; (b) genetically engineer transplant cells to augment their repair potential; and (c) safely target cells to sites of pathology. Here, we review the evidence that magnetic nanoparticles (MNPs) are a ‘*multifunctional nanoplatform*’ that can aid in safely addressing these translational challenges in neural cell/OPC therapy: by facilitating real-time and post-mortem assessment of transplant cell biodistribution, and biomolecule delivery to transplant cells, as well as non-invasive ‘*magnetic cell targeting’* to injury sites by application of high gradient fields. We identify key issues relating to the standardization and reporting of physicochemical and biological data in the field; we consider that it will be essential to systematically address these issues in order to fully evaluate the utility of the MNP platform for neural cell transplantation, and to develop efficacious neurocompatible particles for translational applications.

**Electronic supplementary material:**

The online version of this article (doi:10.1186/2052-8426-2-23) contains supplementary material, which is available to authorized users.

## Introduction

### OPC transplantation therapies for regenerative neurology

**Figure 1:**
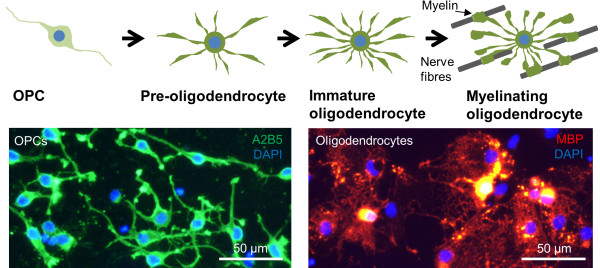
**Schematic diagram illustrating the developmental stages of the oligodendroglial lineage.** Oligodendrocyte precursor cells (OPCs) are proliferative cells which generate myelinating oligodendrocytes, as shown. The insets show typical OPCs (A2B5^+^) and oligodendrocytes (MBP^+^) derived from primary rat cultures (cell culture and immunostaining protocols are detailed in Additional file 1).

Oligodendrocyte precursor cells (OPCs) are proliferative, stem-like cells of the central nervous system (CNS) that have emerged as a key transplant population to promote repair of myelin (the protective, fatty insulating sheath around nerve fibers) [1]. Myelin damage is a key contributor to the pathology of Multiple Sclerosis and spinal cord injury (SCI) [2–5]. The regenerative properties of OPCs are largely due to their capacity to generate oligodendrocytes, the cells that form myelin around nerve fibers [1] (Figure 1), but some evidence suggests that these cells may also dampen destructive processes in pathology sites [6].

Transplantation of OPCs derived from a range of cell sources enhances myelin repair in animal models, including extensive myelin genesis and rescue from lethal conditions in dysmyelinating/ hypomyelinating mutant rodents [6–9]. Introduction of human OPCs into newborn *shiverer* mice resulted in extensive myelination, neurological improvement and enhanced survival in ~26% of mice [10]. Givogri *et al.* transplanted primary OPCs into a neonatal mouse model of metachromatic leukodystrophy, a genetic disorder leading to demyelination and extensive loss of oligodendrocytes [11]; transplant populations generated myelinating oligodendrocytes, identifiable one year post-transplantation, with motor function significantly improved compared with controls. Human embryonic stem cell (ESC)-derived OPCs, transplanted into adult rodent models of SCI, demonstrated remyelination and associated improvement in motor function [12]. From a clinical perspective, OPC transplant populations can be derived from numerous sources [13–16], expanded *in vitro* [14–16], have a good preclinical safety record [17] and have been approved for clinical trial (Geron Corporation, California; GRNOPC1 cells; phase I clinical trial for transplantation of human ESC-derived OPCs into acute SCI [17–19]). This trial recruited 5 of the 10 patients originally intended [20], but has now stopped enrolling, a decision taken by Geron on financial grounds [18, 21–23]. No adverse effects have been reported within one year of transplantation, and a US clinical trials database now lists this study as ‘complete’ (http://www.clinicaltrials.gov, trial identifier NCT01217008, accessed 01 July 2014) [18, 22]. Patients will be followed-up at both 5 and 15 years post-transplantation [20]. Through a deal with BioTime, Asterias Biotherapeutics have acquired the GRNOPC1 stocks (renamed AST-OPC1) and ‘plan to seek FDA clearance to reinitiate human clinical trials’ (asteriasbiotherapeutics.com/our-clinical-focus/opc1/, accessed 01 July 2014) [24]. In a review of 24 preclinical OPC transplant studies for SCI models, no instances of teratomas, systemic toxicity, allodynia, increased mortality or allogeneic immune responses were recorded [17].

**Figure 2:**
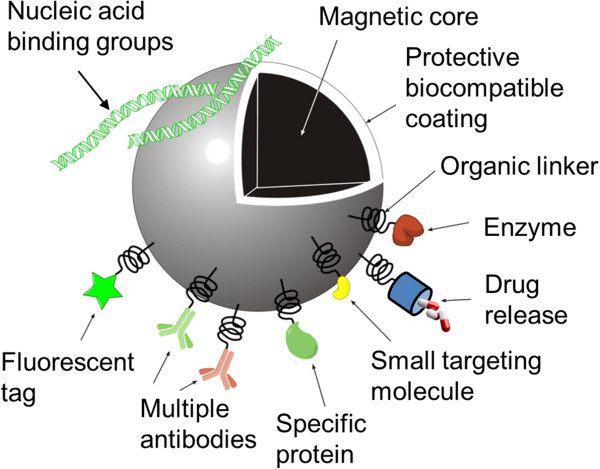
**Schematic diagram illustrating possible MNP features.** Iron oxides (typically magnetite, Fe_3_O_4_, or maghemite, γ-Fe_2_O_3_) provide contrast for MRI and confer ‘superparamagnetism’ to the final particle. A protective biocompatible coating may be functionalized to carry drugs, cell targeting molecules, fluorophores for histological detection and/or nucleic acids for gene delivery.

While such progress in OPC transplantation is highly promising, neural cell therapies still face a number of technical issues/hurdles with respect to testing their efficacy for clinical translation. Here, we review the evidence that magnetic nanoparticles (MNPs) have high utility as multifunctional tools in addressing key challenges in OPC transplantation therapies, most notably in cell tracking. The term MNP encompasses physicochemically diverse synthetic particles, the common element being a magnetic component (Figure 2). This review will focus on particles containing the most widely used magnetic material, iron oxides - these have a good safety profile, with some formulations receiving approval for clinical applications: e.g. ferucarbotran (Resovist), Feridex (Endorem), Ferumoxsil (Lumirem/Gastromark) and Ferumoxtran-10 (Combidex/Sinerem) as MRI contrast agents [25]; NanoTherm for hyperthermic tumor therapy [26]; ferumoxytol (Feraheme) for iron-deficiency anemia [27].

## Review

### The MNP platform can address key challenges confronting OPC transplantation therapies

For neural cell therapies, non-invasive tracking of transplanted cells is essential to correlate functional neurological recovery with transplant cell biodistribution [28]. Further, post-mortem histological analyses are required to assess transplant cell survival, rejection, differentiation profiles and integration, including the extent of myelin genesis. MNPs have been shown to be broadly suitable for both non-invasive and histological imaging, serving as contrast agents for MRI and being readily detectable in post-mortem tissue [29–34]. MRI offers critical advantages for non-invasive imaging including (a) detailed anatomical imaging of inflammation, demyelination/remyelination and assessment of lesion size [35], in parallel with transplant cell detection [26]; (b) lack of potentially harmful radiation (in contrast to CT and PET scanning [28]); and (c) existence of significant infrastructure and expertise in place at clinics worldwide. MNPs provide MRI contrast for imaging through high magnetic moments, which disturb local magnetic field homogeneities [36], resulting in short relaxivity times in water protons in the immediate vicinity of the particles and loss of signal in T2*-weighted MRI images [35, 37, 38]. The contrast generated is proportional to the magnetization of the metal, and inversely proportional to the distance between metal and water protons, so particles designed with high iron content and/or iron near their surface are likely to provide enhanced contrast [26, 37]. Clinical MRI scanners have a resolution of ~500 μm, but high magnetic field (up to 9 T) research scanners have demonstrated a resolution of ~10 μm [26] (although these are unlikely to be safe for human clinical use), with recent refinements allowing for the identification of individual transplant cells [39]. It should be pointed out here that MRI cannot distinguish between intracellular and extracellular MNPs, and dead/dying MNP-labeled cells can therefore provide false-positives [40]. In order to address this confounding issue, studies have correlated MRI contrast with the presence of transplanted cells by post-mortem analyses such as immunostaining. Other methods, such as spatial correlation of MNPs with transplant cell-associated transgene expression or myelin production have also been used to unambiguously identify MNP-labeled OPCs within host tissue.

OPCs can be labeled for imaging applications with physicochemically diverse MNPs, but there is significant inconsistency within the literature in respect of experimental methodologies, particle design/characterization, and outcome measures – leaving doubt regarding the particle properties required to achieve optimal cell labeling. For example, Bulte *et al.* (1999) reported that CG4 cells (an oligodendroglial cell line) did not exhibit MNP-labeling when incubated with dextran-coated MNPs, although the authors report that *“significant”* MNP-labeling of these cells was achieved when the same particles were conjugated with anti-transferrin-receptor antibodies (no numerical data were reported in this study regarding the extent of cellular labeling; Table 1) [29]. When transplanted into spinal cord of myelin deficient (*md*) rats and normal littermates, these cells migrated up to 8.4 mm from the injection site (over 14 d), with *ex vivo* MRI signal correlating well with iron staining and new myelin. In contrast, Franklin *et al.* (1999) successfully labeled >60% of CG4 cells using a dextran-coated MNP without specific cell targeting strategies [30]. These cells were detected *ex vivo* seven days post-transplantation into adult rat ventricles. Frank *et al.* (2003) investigated MNP uptake in CG4 cells using the clinically-approved formulation Feridex (dextran-coated iron oxide particles [41–43]) with and without a complexed transfection agent (Lipofectamine Plus or poly-L-lysine, PLL) [33]. Labeling with unfunctionalized MNPs was reportedly “*low*” (not detectable using Perls’ Prussian blue iron stain), consistent with Bulte *et al.*’s (1999) study, but the cells were successfully labeled using both transfection agents. PLL-functionalized Feridex MNPs were also used by Lepore *et al.* (2006) who reported that *“large numbers of Feridex particles were taken up”* by transgenic OPCs co-cultured with *“neuronal-restricted precursor cells”*; however these co-cultures were uncharacterized and OPC-specific labeling was not quantified [32]. Five weeks post-transplantation into adult rat spinal cord, these cells were detected using *ex vivo* MRI demonstrating migration (up to 5 mm), with good correlation between MRI contrast, iron staining and transgene expression [32]. From these studies, there is insufficient data to reach conclusions regarding the potential physicochemical basis for the different labeling results obtained with dextran coated MNPs in OPCs, as properties such as size and zeta potential differ substantially between the studies, or are entirely unreported.

**Table 1:** **Comparative data from MNP studies involving OPCs or oligodendroglial cell lines**

Ref	Particle	Core (nm ± SD)	Surface	Size (nm ± SD)	Zeta (mV ± SD)	Cell type	Uptake/Labeling/Transfection [incubation conditions]	Transplant	MRI	Toxicity/comments
[29]	MION-46 L (CMIR, USA)	EM: 4.6 ± 1.2; maghemite or magnetite	Dextran	DLS: 8-20	-2.0 ± 0.4 (H_2_O) [44]	CG4	None [48 h; 50–500 μg Fe/ml]	n/a	n/a	No data supplied
	MION-46 L-OX-26		Dextran + anti-Tfr antibody OX-26^c^	Not tested	Not tested		*“numerous intracellular vesicles”*, not quantified [48 h; 2–50 μg Fe/ml]	Myelin deficient (*md*) rat spinal cord, P7.	Post-mortem excised spinal cord, 14 d; MRI contrast correlated well with iron-staining and new myelin	Trypan blue assay: similar viability for labeled/unlabeled cells
[30]	SPIO^a^	2-7^d^	Dextran	Not tested; <400^e^	Not tested	CG4	>60% of cells labeled [24 h; 2 μg Fe/ml]	Adult rat ventricles	Labeled cells detected, post-mortem excised brain, 7 d	No data supplied
[45]	MD-100^a^	EM: 7-8; maghemite or magnetite crystals; multiple per particle	Carboxylated dendrimers	SEC: 20-30 [46]	*Not tested; “highly polarized”*; carboxylation implies negative	Primary rat NSC-derived OPCs (*LacZ* ^+^)	*“remarkable high degree of intracellular labeling”*; within vesicles/endosomes; relaxometry: 9.3 ± 4.3 pg Fe/cell; Ferrozine: 8.5 ± 2.0 pg Fe/cell [48 h; 25 μg/ml]	Long-Evans Shaker (*les*) rat ventricles, P0	I*n vivo*, 6 weeks post-transplantation; *‘excellent’* MRI contrast correlation with *LacZ* expression post-mortem	Labeled cells viable. No difference in growth between labeled/unlabeled cells
[31]	MD-100^a^					CG4	10 pg Fe/cell (control: 1 pg); retained at 1 week in vitro [24–48 h; 10–25 μg Fe/ml]	n/a	n/a	Proliferative capacity and viability unaffected
						Primary rat NSC-derived OPCs (*LacZ* ^+^)	10 pg Fe/cell; retained at 1 week *in vitro* [24–48 h; 10–25 μg Fe/ml]	Long-Evans Shaker (*les*) rat ventricles, P0	I*n vivo*, 6 weeks post-transplantation; *‘excellent’* MRI contrast correlation with *LacZ* expression post-mortem	Proliferative capacity and viability unaffected
[33]	Feridex (Berlex, USA)	5; iron oxide	Dextran	DLS: 50-180	-31.3 (H_2_O)	CG4	“*low*” [48 h; 25 μg Fe/ml]	n/a	Labeled cells detected in gelatin	No data supplied
	Feridex + Lipofectamine		Dextran + Lipofectamine Plus	Not tested	Not tested		14.7 ± 1.7 pg Fe/cell (control: 1.9 ± 0.9) [48 h; 25 μg Fe/ml]			
	Feridex + PLL		Dextran + PLL	Not tested	Not tested		3.8 ± 1.2 pg Fe/cell [48 h; 25 μg Fe/ml]			
[32]	Feridex (Berlex, USA) + PLL	5; iron oxide	Dextran + PLL	DLS: 50-180	-31.3 (H_2_O)	Primary rat GRP + NRP (transgenic)	*“large numbers of particles were taken up”*; localized to endosomes, not nuclear; [48 h, 25 μg Fe/ml]	Adult rat spinal cord	Labeled cells detected, post-mortem excised spinal cord, 5 weeks post-transplantation; 5 mm migration. MNPs correlated well with iron-staining and transgene expression.	Transplanted cells differentiate comparably to unlabeled cells. Labeled transplants elicited greater immune response.
[47]	Fe-NP^a^	5-20; maghemite	Citrate	Not tested	Not tested	OLN-93	159 ± 34 nmol Fe/mg protein, ~2.2 pg Fe/cell^f^ (control: 10 ± 2, ~0.1 pg Fe/cell^f^); concentration-dependent; in intracellular vesicles [48 h; 300 μM]	n/a	n/a	No effects on viability, morphology or proliferation. No Fe leaching from MNPs.
[48]	D-IONP^a^	5-20; iron oxide	DMSA	DLS: 60	-26 ± 3 (FCS^-^)		4200 nmol Fe/mg protein, ~57 pg Fe/cell^f^ (control: 7, ~0.1 pg Fe/cell^f^); concentration-dependent; retained at 24 h [8 h; 4 mM Fe]	n/a	n/a	Concentration-dependent: altered morphology, increased ROS, decreased GSH, but all reversible and viability unaltered.
[49]	D-IONP^a^						957 nmol Fe/mg protein, ~13 pg Fe/cell^f^ (control: 5, ~0.1 pg Fe/cell^f^); decreased to ~620 nmol Fe/mg at 48 h, ~8 pg Fe/cell^f^; concentration-dependent; perinuclear accumulation [24 h; 1000 μM; 55 μg Fe/ml]	n/a	n/a	None evident. No ROS increase. Increased ferritin.
[50]	Neuromag (OZ Biosciences, France)	Not tested; ~0.5% Fe^b^	Not tested; proprietary	DLS: ~216 [51]	Not reported; proprietary	Primary culture-derived OPCs	~21% of cells transfected [oscillating magnetic field; 24 h]	*Ex vivo*, onto organotypic neural tissue slice	n/a	None evident by morphology or cell counts. ‘Transplanted’ cells proliferated, differentiated, integrated into slice.
[52]	Sphero (Spherotech, USA)	Not tested; Polystyrene, nile red-stained	Carboxylated Fe_3_O_4_/ polystyrene; 15-20% Fe^b^	EM: 200–390 (mean 360);^b^ DLS: 843-961	-14.3; -23.13^b^		~60% of cells labeled; heterogeneous extent, typically ‘low’. Time- and concentration-dependent. [24 h; 50 μg/ml]	n/a	Particles in agar gel show concentration-dependent contrast	None evident by morphology or cell counts. Generated MNP-labeled oligodendrocytes. Intracellular MNPs appear stable.
[53]	Fe_3_O_4_-PEI-RITC^a^	EM: 24.3 ± 5.7; XRD: 25.5; Fe_3_O_4_; ~58% Fe [54]	1800 MW PEI; RITC [54]	Not tested	+18.6 [54]		~50% [5 μg/ml], ~60% [24 h; 20 μg/ml]. Concentration-dependent.	n/a	Particles show concentration-dependent contrast	None evident by morphology or cell counts
[55]	D-IONP^a^	EM: 4-20	DMSA	DLS: 53 (H_2_O); 52 ± 2 (medium, FCS^-^)	-58 ± 4 (H_2_O); -20 ± 10 (medium, FCS^-^)	OLN-93	Specific iron: ~1700 nmol/mg protein, ~23 pg Fe/cell^f^ (~30-50% represents extracellular MNPs; control: 69 nmol/mg, ~1 pg Fe/cell^f^) [FCS^-^; 4 h, 1 mM]	n/a	n/a	Unaltered LDH activity
				DLS: 109 ± 23 (medium, FCS^+^)	-9 ± 1 (medium, FCS^+^)		201 ± 63 nmol/mg protein, ~3 pg Fe/cell^f^ [FCS^+^]			
	BP-D-IONP^a^	EM: 4-20	DMSA + BODIPY	DLS: 63 (H_2_O); 61 ± 5 (medium, FCS^-^)	-58 ± 18 (H_2_O); -28 ± 2 (medium, FCS^-^)		Specific iron: ~1800 nmol/mg protein, ~24 pg Fe/cell^f^ (~30-50% represents extracellular MNPs; control: 69 nmol/mg, ~1 pg Fe/cell^f^); Not lysosome-associated. [FCS^-^; 4 h, 1 mM]	n/a	n/a	Unaltered LDH activity
				DLS: 138 ± 24 (medium, FCS^+^)	-10 ± 1 (medium, FCS^+^)		171 ± 15 nmol/mg, ~2 pg Fe/cell^f^ [FCS^+^]			

^a^In-house synthesis; ^b^Manufacturer supplied data; ^c^Internalizing anti-transferrin receptor monoclonal antibody; ^d^Based on patent PCT/JP93/001092; ^e^Not reported, but measurements of electron micrograph in article suggest <400 nm (could be MNP aggregate); ^f^pg Fe/cell values not reported but calculated as per Additional file 1; CG4 = oligodendroglial cell line; DIV = days in vitro; DMSA = dimercaptosuccinic acid; DLS = dynamic light scattering; EM = electron microscopy; FCS = fetal calf serum; GFP = green fluorescent protein; GRP = glial restricted precursor; GSH = glutathione, antioxidative molecule; LacZ = gene encoding *β*-galactosidase; LDH = lactate dehydrogenase; MBP = myelin basic protein, oligodendrocyte marker; NRP = neuronal restricted precursor; NSC = neural stem cell; OLN-93 = oligodendroglial cell line; OPC = oligodendrocyte precursor cell; PEI = polyethyleneimine; PLL = poly-L-lysine; RITC = rhodamine B isothiocyanate; ROS = reactive oxygen species; SEC: size exclusion chromatography; Tfr = transferrin receptor; XRD = powder X-ray diffraction.

The Bulte group reported comparable uptake levels in CG4 cells, OPCs and other cell types, concluding that MNP-uptake is non-specific and independent of cell type [31, 45]. However, our group has reported substantial variability in MNP-uptake dynamics between neural cell types [52]. Concentration- and time-dependent uptake of carboxylated polystyrene MNPs was shown for four neural cell types (microglia, astrocytes, OPCs and oligodendrocytes) derived from primary cultures. Up to 60% of OPCs were labeled, with heterogeneity in the extent of MNP-loading. Notably, microglia exhibited very avid and extensive MNP uptake compared with the other cell types, with oligodendrocytes demonstrating the lowest levels of uptake [52].

Hohnholt *et al.* (2010, 2011) used MNPs with the goal of studying iron metabolism and toxicity, rather than labeling, in OLN-93 cells (an oligodendroglial cell line) reporting concentration-dependent uptake of both citrate- and dimercaptosuccinic acid (DMSA) coated MNPs (up to 300-fold increases in average intracellular iron) [47–49]. In a subsequent study, Petters *et al.* (2014) functionalized these DMSA-coated MNPs with a fluorophore and demonstrated uptake comparable to particles lacking conjugated fluorophores (69 nmol Fe/mg cellular protein control; ~1700 nmol/mg without fluorophore; ~1800 nmol/mg with fluorophore; to aid comparisons with other studies, we have re-calculated these values, as described in Additional file 1; respectively, these values are ~1 pg Fe/cell, ~23 pg Fe/cell and ~24 pg Fe/cell) [55]. Importantly, the authors characterized these particles before and *after* functionalization, an oft-omitted step (Table 1; [29, 33]): size increased by 17%, zeta potential changed from -20 to -28 mV. This study noted nine-fold greater levels of uptake in the absence of serum, compared to serum-supplemented medium, illustrating the influence of the biochemical composition of media on particle-cell interactions.

Many MNPs are readily detected due to their metal content, for example by simple histochemical iron staining, which in turn correlates well with MRI observations of MNP-labeled OPCs post-transplantation [29, 32]. For particles not amenable to metal-based detection (e.g. due to low iron content), fluorophores can be incorporated, either internally or attached to the particle surface, facilitating post-mortem detection by fluorescence imaging. For example, Kircher *et al.* demonstrated detection of a cyanine dye (Cy5.5)-tagged dextran-coated MNP through fluorescence microscopy of post-mortem tissue, although this particle was used to delineate a brain tumor, rather than to track a transplant population [56].

Long-term tracking of transplanted cells is highly dependent upon label retention, but dilution of MNP-labeling has been observed *in vitro* and *in vivo*, being attributed at least in part to cell proliferation [29, 40]. This represents a particular challenge for imaging the biodistribution/migration of proliferative populations such as OPCs. Although MNP retention by OPCs has been reported for 7 d *in vitro* and 6 weeks post-transplantation (upper limits not determined) [31, 45], no studies have systematically quantified proliferative dilution of MNPs, or distinguished between particle loss due to cell proliferation versus cellular excretion by exocytosis. A further concern is whether particles are retained during differentiation into mature oligodendrocytes, as the primary goal of OPC therapy is to replace lost/damaged oligodendrocytes [1, 5]. Therefore, the ability to image these differentiated cells long-term in areas of regeneration is key for myelinating therapies. Oligodendrocytes are post-mitotic cells, therefore particle loss due to proliferative dilution is eliminated. Indeed in our experiments, when pulse-labeled OPCs were subsequently differentiated and maintained for 30 days, a significant proportion (>50%) of oligodendrocytes displayed MNP-labeling, suggesting that the differentiated progeny can *‘inherit’* MNPs and retain the label for long-term imaging [53].

### MNPs have promising safety profiles in OPCs

In order to develop MNPs for clinical cell therapies, it is of paramount importance to assess their potential cytotoxic effects in neural transplant populations. Oligodendroglial cells contain more iron than any other CNS cell type, but are also the most vulnerable to excess iron, which typically leads to oxidative stress due to reactive oxygen species (ROS) [49]. It is of note that oxidative stress has been linked with oligodendrocyte damage in diseases such as Multiple Sclerosis [57, 58], indicating that MNP-induced genesis of ROS could be similarly deleterious to labeled transplanted oligodendroglial cells. In other neural cells, MNPs have been shown to impair cellular function through mechanisms including disruption to the cytoskeleton/cell membrane [59, 60] or intracellular trafficking processes [61, 62], and direct damage to intracellular organelles [61] including by iron release during particle degradation [63]. Through these or other mechanisms, MNP uptake could also perturb cellular behavior, including capacity for migration or proliferation [64].

The Dringen group have used the OLN-93 oligodendroglial cell line to conduct the most detailed MNP-OPC toxicity studies to date, including demonstration of uptake of citrate-coated MNPs without affecting viability, morphology or proliferation, and without evidence of iron leaching [47]. Ferritin was greatly upregulated in response to increased Fe levels, storing Fe in a redox inactive form and protecting against iron-related toxicity [47]. A battery of assays found no evidence of acute cytotoxicity (72 h) for DMSA-coated MNPs [49]. For the same MNPs and cells, another study reported morphological changes, decreased glutathione (an antioxidative molecule) and increased ROS, but these changes were reversible and did not affect viability [48, 65]. Consistent with these data, OPCs labeled with other MNPs are generally reported as having viability and behavior comparable to unlabeled OPCs (Table 1; [29, 31, 32, 45, 52, 53, 55]).

### Combinatorial therapies and OPC transplantation: using multimodal MNPs to achieve multiple therapeutic goals

While cell therapy alone is demonstrably efficacious, a widely-held view in the regenerative neurology community is that ‘*combinatorial*’ therapies (e.g. cell transplantation plus drug/gene delivery) achieve more impactful clinical regenerative outcomes than single therapeutic strategies [66–70]. For example, transplanting OPCs genetically engineered to secrete neurotrophic factors showed significantly greater improvement in SCI injury models than transplanting unmodified OPCs, or fibroblasts secreting the same neurotrophins [68, 71]. A major translational challenge currently is to achieve safe and effective genetic engineering of transplant populations. We have shown that MNPs can deliver both reporter and therapeutic genes to OPCs, a process significantly enhanced by the use of state-of-the-art ‘*magnetofection*’ strategies (applied static or oscillating magnetic fields to enhance particle-cell contact; up to 21% transfection efficiency in OPCs derived from primary sources) [50]. In contrast to the precursor cells, differentiated oligodendrocytes showed far lower transfection levels (up to 6%), suggesting that the proliferative or endocytotic properties of the OPCs may make these cells relatively amenable to MNP-mediated transfection compared with their progeny [72]. As far as we are aware, these are the only reports of MNP-mediated gene delivery to cells of the oligodendrocyte lineage available.

A further translational challenge is achieving targeted delivery of transplant cells to lesions while limiting secondary pathology. Spatial manipulation of MNP-labeled cells has been demonstrated using external magnetic fields – a technique that could retain/localize transplant cells at target sites by magnetic cell *‘capture’* following intravenous/intrathecal delivery, of high relevance in situations where a limited cell source exists. For example, an implanted magnet localized (limited dispersion of) MNP-labeled cells at a rat spinal cord lesion site following intrathecal delivery of mesenchymal stem cells [73] and bone marrow stromal cells [74]. Magnetic fields have also been used to localize MNP-labeled cells at a specific region of the retina following intravitreal injection (reportedly ~360000 transplanted cells, compared to ~10000 cells without applying a magnet), and following intravenous delivery of cells (~42000 cells, compared to ~4000 cells without applying a magnet) [75]. Magnetic cell targeting has not yet been demonstrated for OPCs, but may be feasible for SCI as above. Furthermore, the superparamagnetic properties of particles used for cell labeling (i.e. where particle magnetic properties are exhibited only in the presence of a magnetic field) can help overcome issues of cell aggregation and blockade of capillaries following systemic delivery [76]. In conjunction with the imaging potential of MNP-labeled transplant populations, the above findings highlight the high potential of MNPs to serve as a *‘multifunctional platform’* to address key challenges in neural cell therapy [54], summarized in Table 2.

**Table 2:** **Challenges for cell transplantation therapies and the relevant utility of magnetic nanoparticles**

	Gene delivery to transplant populations	Non-invasive transplant tracking	Cell targeting/localization	Post-mortem transplant identification
**Clinical needs**	• Therapeutic biomolecule delivery for combinatorial therapies.	• Assess on-target/off-target delivery.	• Deliver high number of cells to lesions.	• Assess survival, differentiation, integration into host.
	• Transgenes more effective than separate biomolecule delivery.	• Correlate clinical improvement/side-effects with cell presence.	• Reduce cell loss/maximize therapeutic effect.	• Correlate biodistribution of cells with evidence of regeneration.
			• Minimize off-target effects.	
**Current methods**	• Viral vectors efficient but raise clinical safety concerns and require substantial infrastructure.	• Plasmonic resonance of gold nanoparticles: promising, but little infrastructure; gold particles cannot be non-invasively manipulated.	• Invasive injection into lesion parenchyma risks secondary damage.	• Dyes frequently leak and label host cells.
	• Many nonviral methods inefficient, unsafe and/or not clinically relevant.	• Radiation exposure is associated with CT scans (X-rays) and PET scans (tracers).	• Distal intravenous/intrathecal delivery limits adherence/accumulation at target.	• *LacZ* transgene expression confounded by host microglial *β*-galactosidase activity.
			• Cell-seeded scaffolds require invasive delivery at lesion site.	• Mismatched gender/species/mutant transplants are not clinically relevant.
**Benefits of MNPs**	• Comparable efficiency to other nonviral systems.	• Provide contrast for non-invasive MRI.	• Non-invasive manipulation of MNP-labeled cells using magnetic fields for:	• Provide MRI contrast.
	• Safe protocols developed.	• Clinical MRI equipment and expertise widely available.	• Retention of cells at target site, facilitating adhesion.	• Metals (e.g. iron) can be stained.
			• ‘Capture’ of cells from blood/cerebrospinal fluid; safe delivery distal to lesion.	• Fluorophores can be incorporated into MNPs (for preclinical testing).

**Figure 3:**
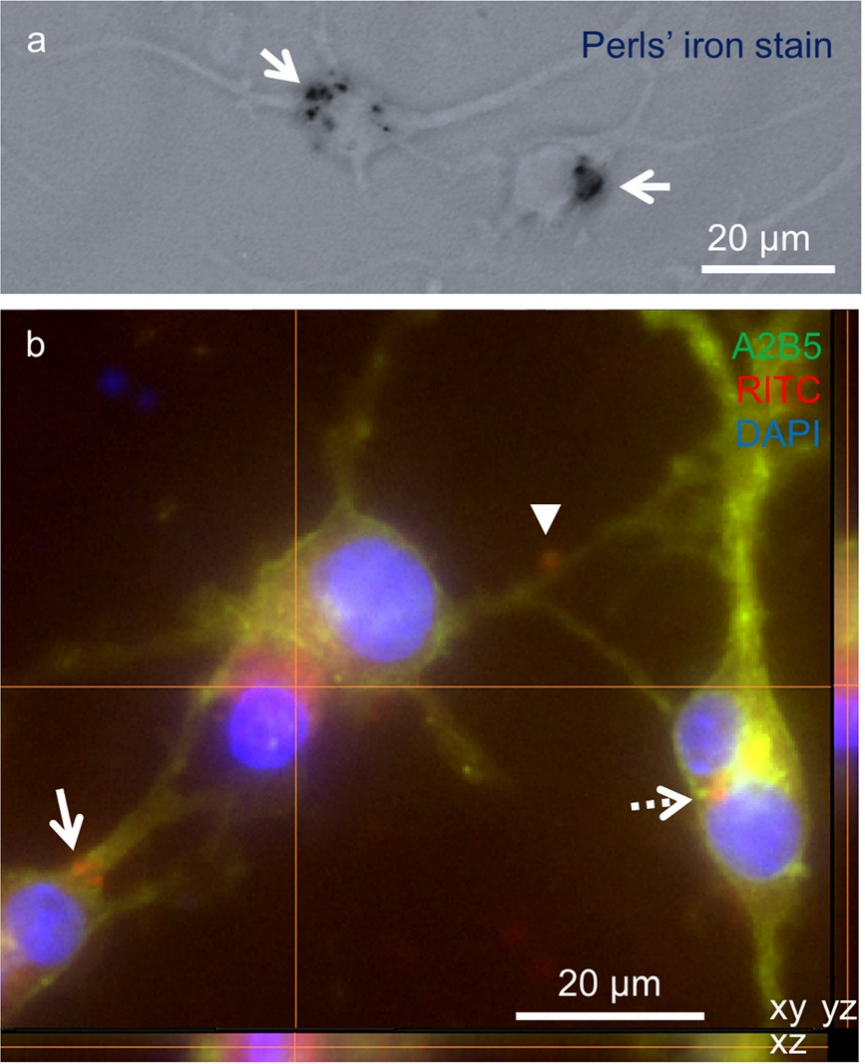
**OPCs can be labeled with a multimodal MNP.** ~60% of OPCs derived from a primary source exhibit uptake of a multifunctional MNP. **(a)** Phase contrast micrograph showing Perls’ iron staining of MNPs (arrows). **(b)** Z stack fluorescence micrograph confirming intracellular presence of MNPs, both perinuclear (crosshairs and dashed arrow) and cytoplasmic (arrow). Arrowhead indicates extracellular particle accumulation. OPCs were derived from primary rat cerebral cortex cultures and plated 24 h before MNP incubation: Fe_3_O_4_-PEI-RITC MNPs, 20 μg/ml, 24 h [54]. Cultures were then fixed (4% paraformaldehyde) and immunostained [54]. Further details can be found in Additional file 1. Particle characteristics are detailed in Table 1. A2B5 is an OPC marker. RITC = rhodamine B isothiocyanate.

However, it should be noted that the overwhelming majority of MNPs described so far for neural applications have been *unimodal*. Rapid advances in materials chemistry in recent years have led to the development of a spectrum of complex, multimodal MNPs which simultaneously mediate multiple functions [54]. For cell therapies, purpose designed *multimodal* MNPs could mediate cell imaging, genetic modification and magnetic cell targeting. One such multimodal MNP was recently described, with high iron content for MRI contrast (and possibly cell targeting), a fluorophore for histological detection, and potential for gene delivery which was demonstrated in astrocytes – a major neural transplant population [54]. The particles were synthesized using a chemical grafting procedure to link polyethyleneimine (PEI) covalently to the surface of Fe_3_O_4_ MNPs. This methodology allowed for permanent linking of the PEI to the MNP surface, of benefit for use in biological fluids with high electrolyte concentrations; this also enabled overall particle size to be restricted to <50 nm and resulted in high iron content for the particles (*ca* 65% by weight), of potential benefit for imaging and magnetic targeting applications [54]. A red dye (rhodamine B isothiocyanate, RITC) was then bound to the PEI skeleton, with the final particles being denoted Fe_3_O_4_-PEI-RITC. The particles could be imaged using standard fluorescence/confocal microscopy and MRI, and were compatible for use with a range of histological methods [54]. Further, the chemical design of the particles also allowed for high versatility with respect of the use of other functional polymers and binding chemistries for nanoparticle functionalization, therefore particles of greater functional complexity can be evolved from this basic prototype. Using a simple one-step procedure, we have found that ~60% of OPCs could be safely labeled with these novel MNPs (Figure 3, previously unpublished data; particle properties can be found in Table 1, ref [53]), highlighting the therapeutic potential of multimodal particles in OPC transplantation [53]. Whilst our previous work showed that the gene delivery capacity of the particles was low overall (<1%), we consider that with further work directed towards enhancement of their transfection potential, such particles can prove a valuable ‘theragnostic’ tool for the developmental testing and clinical translation of neural cell therapies for regenerative neurology [54].

## Conclusions

### Biological perspectives: the need for standardization of reporting

The MNP platform offers high promise for neural transplantation applications, but the field is still in its relative infancy. In-depth and cross-disciplinary studies between materials chemists and transplantation neurobiologists are required to fully evaluate MNPs as an adjunct tool for OPC transplantation. For example, despite the key advantages offered by multimodal MNPs for OPC transplantation, there is a critical lack of neurocompatible and multimodal MNPs, representing a major scientific and commercial gap. The potential for magnetic cell targeting of OPCs to injury foci has never been assessed, and the processes of proliferative dilution and particle *‘inheritance’* by daughter oligodendrocytes are poorly understood. Further, much of the research investigating MNP uptake and handling by OPCs has relied on cell lines, whose behavior can differ markedly from primary cells – consequently, biological data derived from cell lines may have limited predictive value. For example, Pinkernelle *et al.* report six-fold greater MNP-labeling in the *‘neuron-like’* cell line PC12 than in primary neurons [51]; similar comparative analyses are required for OPCs.

The standardization of data reporting from MNP-labeling studies is essential to guide advances in nanoparticle synthesis and design. As with many biomaterials studies, MNPs used for OPC labeling are typically not fully characterized, yet these details are essential to identify parameters relevant for improving biomaterial design. There has been little systematic attempt to correlate MNP physicochemical properties with extent of OPC labeling (Table 1), of high relevance from a cell therapy perspective. Findings regarding the ability of oligodendroglial cells to take up MNPs without conjugated targeting molecules/transfection agents are contradictory (e.g. Bulte [29] and Frank [33] versus Franklin [30], all using dextran-coated MNPs); the reasons underpinning these differences are difficult to address in the absence of detailed particle characterization. Typically, reports should include size, shape and surface charge/functionalities of the *final* particle, measured within physiologically relevant media. The evaluation of OPC interactions with MNPs possessing a wider range of physicochemical properties can inform the tailored development of MNPs for specific transplantation applications. Such investigations should ideally include ultrastructural analyses of particle-cell interactions, along with evaluations of intracellular handling and particle fate to establish cellular processing mechanisms for different particles. This information can guide the development of MNPs with potential for endosomal escape, or suggest specific uptake mechanisms to which MNPs should be preferentially targeted for optimal labeling.

Other substantial knowledge gaps are apparent from the literature. Few studies report the proportions of OPCs exhibiting MNP-labeling, or conduct assessments of the extent of MNP-loading and its correlation with imaging capacity. More often, researchers provide an average iron content per cell measurement, which will mask any heterogeneity of particle accumulation within a cell population. This is particularly relevant to primary populations (the most likely cell source for transplantation therapies) which show considerable heterogeneity in behavior including particle uptake [52], unlike cell lines which behave in a relatively clonal manner [77]. Most studies report limited MNP-associated cytotoxicity in OPCs, but generally without *numerical* viability/safety data, a significant shortcoming as this information is vital to developing biocompatible particles and safe labeling protocols. Microarray/proteomic analyses are essential for detailed molecular analyses of MNP toxicity, particularly the long term safety of transplant populations. This should progress in parallel with functional assays of the regenerative capacity of transplanted MNP-labeled OPCs (e.g. cell migration and myelin genesis). It can be predicted that such work can facilitate the development and application of this platform technology to neural cell therapies, in order to promote repair mechanisms following neurological pathology – currently a key goal for regenerative medicine globally.

## Electronic supplementary material

Additional file 1: **Supplementary methods, detailing culture and immunostaining protocols, and calculations for converting nmol Fe per mg cellular protein to pg Fe per cell.** (PDF 164 KB)

## Abbreviations

CG4: Oligodendroglial cell line
CNS: Central nervous system
DMSA: Dimercaptosuccinic acid
ESC: Embryonic stem cell
MBP: Myelin basic protein
MNP: Magnetic nanoparticle
OLN-93: Oligodendroglial cell line
OPC: Oligodendrocyte precursor cell
PEI: polyethyleneimine
PLL: Poly-L-lysine
RITC: Rhodamine B isothiocyanate
ROS: Reactive oxygen species
SCI: Spinal cord injury.
