# Supplementary material for: Magnetic nanoparticles for oligodendrocyte precursor cell transplantation therapies: progress and challenges
Source: Mol Cell Ther. 2014 Jul 28;2:23. doi: 10.1186/2052-8426-2-23 (PMC4452053; doi:10.1186/2052-8426-2-23)
Supplement: Supplementary file 1 — Additional file 1: Supplementary methods, detailing culture and immunostaining protocols, and calculations for converting nmol Fe per mg cellular protein to pg Fe per cell. (PDF 164 KB) [file 40591_2014_27_MOESM1_ESM.pdf]

Additional File 1: Supplementary methods – Molecular and Cellular Therapies

Magnetic Nanoparticles for Oligodendrocyte Precursor Cell Transplantation Therapies: Progress and Challenges

Stuart Iain Jenkins, Divya Maitreyi Chari, Keele University

Humphrey Hak Ping Yiu, Heriot-Watt University

Matthew Jonathan Rosseinsky, University of Liverpool

These methods describe the culture and staining protocols used to obtain the micrographs in Figures 1 and 3 (adapted from [1]), and also detail calculations used to estimate the mass of iron per cell from data presented as nmol Fe/mg cellular protein.

The care and use of animals was in accordance with the Animals (Scientific Procedures) Act of 1986 (United Kingdom) with approval by the local ethics committee.

Reagents and equipment:

Tissue culture-grade plastics, media, and media supplements were from Fisher Scientific (Loughborough, UK) and Sigma-Aldrich (Poole, UK). Recombinant human platelet-derived growth factor (PDGF-AA) and basic fibroblast growth factor (FGF2) were from Peprotech (London, UK). Monoclonal rat anti-MBP was from Serotech (Kidlington, UK), monoclonal mouse anti-A2B5 and chemicals for Perls' staining (potassium hexacyanoferrate and HCl) were from Sigma-Aldrich (Poole, UK), and secondary antibodies [Fluorescein isothiocyanate (FITC)- or Cy3-conjugated] were from Jackson ImmunoResearch Laboratories Inc. (West Grove, PA, USA). Mounting medium (with and without DAPI, 4',6-diamidino-2-phenylindole) was from Vector Laboratories (Peterborough, UK).

Oligodendrocyte precursor cell (OPC) cultures:

Primary mixed glial cultures were prepared from dissociated cerebral cortices of postnatal day 1 – 3 Sprague-Dawley rats, based on an established protocol [2]. Cultures were maintained in D10 medium [Dulbecco's modified Eagle's medium (DMEM) supplemented with 10% fetal bovine serum, 2 mM glutaMAX-I, 1 mM sodium pyruvate, 50 U/ml penicillin, and 50 µg/ml streptomycin] at 37°C in 5% CO2/95% humidified air for 8 – 10 days, then shaken for 2 h on a rotary shaker at 200 rpm. This medium (containing largely

microglia) was discarded, fresh D10 medium was added and allowed to re-gas, then the flasks were shaken overnight at 200 rpm. The resulting medium, containing largely OPCs, was transferred to non-tissue-culture grade petri dishes, to which microglia readily attach but OPCs do not, reducing residual microglial contamination. After 30 min, unattached cells were resuspended in OPC maintenance medium (OPC-MM: DMEM supplemented with 2 mM glutaMAX-I, 1 mM sodium pyruvate, 10 nM biotin, 10 nM hydrocortisone, 30 nM sodium selenite, 50 µg/ml transferrin, 5 µg/ml insulin, 0.1% bovine serum albumin, 50 U/ml penicillin, 50 µg/ml streptomycin, 10 ng/ml PDGF-AA, and 10 ng/ml FGF2) then plated onto poly-D-lysine (PDL) coated glass coverslips in 24-well plates (0.3 ml/well, at 3 x 10^4 cells/cm^2). OPC cultures were maintained for 24 h before incubation with MNPs, to allow cell adherence and re-growth of processes.

Oligodendrocyte culture:

OPCs were plated in OPC-MM for 24 h, then the medium was replaced with Sato medium [DMEM supplemented with 2 mM glutaMAX-I, 1 mM sodium pyruvate, 1x N2 supplement (insulin, human transferrin, progesterone, putrescin, selenite [3]), 30 nM thyroxine, 30 nM triiodothyronine, 50 U/ml penicillin, and 50 µg/ml streptomycin] to induce differentiation (medium changes every 2 – 3 days). Cultures were fixed and immunostained after 7 days.

Fe_3O_4-PEI-RITC MNPs:

The Fe_3O_4-PEI-RITC particles comprise a magnetite core, surrounded by a covalently attached polyethyleneimine (PEI) layer, onto which a red dye (rhodamine B isothiocyanate, RITC) is bound. The synthesis of these particles and their characterization have been described elsewhere, with TEM analyses indicating a uniform spherical shape and a core diameter of 24.3 ± 5.7 nm [4].

Fe3O4-PEI-RITC uptake experiments:

At 24 h after plating, OPCs were incubated with 20 µg/ml MNPs for 24 h. Samples were washed with phosphate buffered saline (PBS), then fixed and either immunostained, or processed for Perls' Prussian blue histochemical staining.

Immunocytochemistry:

In all cases, washed cells were fixed with 4% paraformaldehyde [PFA; room temperature (RT); 25 min] then washed again (PBS). For staining, cells were incubated with blocking solution (5% serum in PBS, with 0.3% Triton X-100 for MBP; RT; 30 min), then with primary antibody in blocking solution (A2B5 1:200; MBP 1:200; 4°C; overnight). Cells were

then washed with PBS, incubated with blocking solution (RT; 30 min), and incubated with the appropriate FITC- or Cy3-conjugated secondary antibody in blocking solution (1:200; RT; 2 h). Finally, coverslips were washed with PBS and mounted with the nuclear stain DAPI.

Fluorescence microscopy:

Samples were imaged using an Axio Scope A1 fluorescence microscope (Carl Zeiss MicroImaging GmbH, Goettingen, Germany), and the images merged using Adobe Photoshop CS3 (version 10.0.1).

Z-stack fluorescence microscopy:

Z-stack fluorescence micrographs were created using a Nikon Eclipse 80i microscope fitted with a CA742-95 camera (Hamamatsu Photonics, Hamamatsu, Japan), with manual focus stepping at 0.5 µm, and the image manipulations performed using NIS Elements (Nikon, version 3.00).

Perls' Prussian blue iron staining:

Fixed OPCs were incubated with 2% potassium ferricyanide in 2% HCl for 10 min, washed three times with distilled water and then mounted in glycerol-based mounting medium without DAPI.

Calculations to convert data presented as nmol Fe/mg cellular protein to pg Fe/cell:

The Dringen group at Bremen University have published OLN-93 MNP uptake data as nmol Fe/mg cellular protein [5–8]. This is difficult to compare with other studies, which typically report pg Fe/cell. However, this group have previously published data which can be analysed to determine the average quantity of protein per OLN-93 cell [9]. Figure 1, page 139, shows cellular protein content (µg/well) from a 12 well plate (~22.1 mm diameter, ~384 mm^2 area) and number of cell nuclei per mm^2 at 24, 48 and 72 h. These approximate values were derived from the bar graphs in this Figure, and the three timepoints compared (Table S1).

Table S1: Protein per OLN-93 cell

	Timepoint (h)		
	**24**	**48**	**72**
Nuclei per mm^2	435	775	2150
Cells per well (nuclei/mm^2 x 384 mm^2)	166886	297325	824839
Protein (µg/well)	39	78	189
Protein/cell (mg x 10^{-7})	2.337	2.623	2.291

The average value across these timepoints was 2.42 x 10^{-7} mg protein/cell, equating to 4,136,980 OLN-93 cells per mg protein. The Fe data presented as nanomoles per mg cellular protein were converted to pg Fe per 1 mg cellular protein (Fe MW 55.845). By combining these values with the number of cells expected per mg protein, approximate values were derived for pg Fe/cell (Table S2).

Table S2: Conversion of Fe measures from nmol Fe/mg protein to pg Fe/cell (assuming 1 mg protein = 3,968,703 OLN-93 cells)		
**Reported Fe values (nmol/mg cellular protein)**	**Fe (pg per 1 mg protein)**	**Fe (pg/cell)**
5	279225	0.07
7	390915	0.09
10	558450	0.13
69	3853305	0.93
159	8879355	2.15
171	9549495	2.31
201	11224845	2.71
620	34623900	8.37
957	53443665	12.92
1700	94936500	22.95
1800	100521000	24.30
4200	234549000	56.70

References

1. Jenkins SI: **Applications of magnetic particles for oligodendrocyte precursor cell transplantation strategies**. *PhD thesis*. Keele University, Institute for Science and Technology in Medicine; 2013.

2. McCarthy KD, de Vellis J: **Preparation of separate astroglial and oligodendroglial cell cultures from rat cerebral tissue.** *J Cell Biol* 1980, **85**:890–902.

3. Rubio N, Rodriguez R, Arevalo MA: **In vitro myelination by oligodendrocyte precursor cells transfected with the neurotrophin-3 gene.** *Glia* 2004, **47**:78–87.

4. Yiu HHP, Pickard MR, Olariu CI, Williams SR, Chari DM, Rosseinsky MJ: **Fe3O4-PEI-RITC magnetic nanoparticles with imaging and gene transfer capability: development of a tool for neural cell transplantation therapies**. *Pharm Res* 2012, **29**:1328–1343.

5. Petters C, Bulcke F, Thiel K, Bickmeyer U, Dringen R: **Uptake of fluorescent iron oxide nanoparticles by oligodendroglial OLN-93 cells.** *Neurochem Res* 2014, **39**:372–83.

6. Hohnholt M, Geppert M, Dringen R: **Effects of iron chelators, iron salts, and iron oxide nanoparticles on the proliferation and the iron content of oligodendroglial OLN-93 cells.** *Neurochem Res* 2010, **35**:1259–68.

7. Hohnholt MC, Dringen R: **Iron-dependent formation of reactive oxygen species and glutathione depletion after accumulation of magnetic iron oxide nanoparticles by oligodendroglial cells**. *J Nanoparticle Res* 2011, **13**:6761–6774.

8. Hohnholt MC, Geppert M, Dringen R: **Treatment with iron oxide nanoparticles induces ferritin synthesis but not oxidative stress in oligodendroglial cells**. *Acta Biomater* 2011, **7**:3946–3954.

9. Hohnholt MC: **Metabolism of iron and iron oxide nanoparticles in glial cells**. *PhD thesis*. Bremen University, Centre for Biomolecular Interactions; 2011.
